# Using national laboratory data to assess cumulative frequency of linkage after transfer to community‐based HIV clinics in South Africa

**DOI:** 10.1002/jia2.25326

**Published:** 2019-06-27

**Authors:** Ingrid V Bassett, Mingshu Huang, Christie Cloete, Sue Candy, Janet Giddy, Simone C Frank, Kenneth A Freedberg, Elena Losina, Rochelle P Walensky, Robert A Parker

**Affiliations:** ^1^ Division of Infectious Diseases Massachusetts General Hospital Boston MA USA; ^2^ Division of General Internal Medicine Massachusetts General Hospital Boston MA USA; ^3^ Medical Practice Evaluation Center Department of Medicine Massachusetts General Hospital Boston MA USA; ^4^ Harvard University Center for AIDS Research (CFAR) Boston MA USA; ^5^ Harvard Medical School Boston MA USA; ^6^ Biostatistics Center Massachusetts General Hospital Boston MA USA; ^7^ McCord Hospital Durban South Africa; ^8^ Department of Academic Affairs, Research and Quality Assurance Corporate Data Warehouse National Health Laboratory Services Johannesburg South Africa; ^9^ Departments of Epidemiology and Health Policy and Management Harvard T.H. Chan School of Public Health Boston MA USA; ^10^ Departments of Biostatistics and Epidemiology Boston University School of Public Health Boston MA USA; ^11^ Division of Rheumatology Department of Medicine, and Department of Orthopedic Surgery Brigham and Women's Hospital Boston MA USA

**Keywords:** South Africa, National Health Laboratory Service, patient transfers, community‐based clinics, CD4 and viral load data, retention in care, transfer of HIV care

## Abstract

**Introduction:**

Changes to the U.S. President's Emergency Plan for AIDS Relief (PEPFAR) funding have led to closures of non‐governmental HIV clinics with patient transfers to government‐funded clinics. We sought to determine the success of transfers in South Africa using a national data source.

**Methods:**

All adults (≥18 years) on antiretroviral therapy (ART) who visited a single PEPFAR‐funded hospital‐based HIV clinic in Durban, South Africa from March to June 2012 were transferred to community‐based clinics. Previously, we matched patient records from the hospital‐based HIV clinic with National Health Laboratory Services (NHLS) Corporate Data Warehouse (CDW) data to estimate the proportion of patients with a CD4 count or viral load (VL) in the CDW during the year before transfer. As a proxy for retention in care, in this study we evaluated whether patients had a CD4 count or VL at another facility within approximately three years of transfer. Patients referred to a private doctor at transfer were excluded from the analysis. We assessed predictors (age, sex, CD4 count, VL status, ART duration and location of future care) of not having post‐transfer laboratory data using Cox proportional hazards models.

**Results:**

Of the 3893 patients referred to a government facility at transfer, 41% were male and median age was 39 years (IQR 34 to 46). There was a post‐transfer CD4 count or VL from another facility for 23% of these individuals within six months, 44% within one year, 57% within two years and 61% within approximately three years. Male sex (aHR 1.20, 95% CI 1.10 to 1.31) and shorter duration on ART (<3 months, aHR 3.80, 95% CI 2.77 to 5.21; three months to one year, aHR 1.32, 95% CI 1.15 to 1.51, each compared with >1 year) were associated with not having a post‐transfer record.

**Conclusions:**

Using data from the NHLS CDW, 61% of patients had evidence of a post‐transfer laboratory record at another facility within approximately three years after closure of a large South African HIV clinic. Males and those with shorter time on ART prior to transfer were at highest risk for lacking follow‐up laboratory data. As patients transfer care, national data sources can be used to evaluate long‐term patient care trajectories.

## 1 Introduction

South Africa has the largest HIV treatment programme in the world, with more than 3.1 million people on antiretroviral therapy (ART) 1. Because of a gradual, planned funding reduction from the U.S. President's Emergency Plan for AIDS Relief (PEPFAR) to promote transition to full country‐led fiscal responsibility, the South African government has continued to expand its national programme 2, 3, 4, 5, 6. As part of this expansion, patient care has shifted from hospital‐based, doctor‐managed clinics towards nurse‐led, public sector community clinics, resulting in large‐scale patient transfers 7, 8.

The clinical implications of patient transfers are difficult to assess, but have become increasingly important as the South African government focuses on strategies to improve linkage to and retention in HIV care 9, 10. Limited available data show similar clinical outcomes for those transferred to primary health clinics compared with hospital‐based clinics 11, 12, 13. However, these studies reflect outcomes of selected, clinically stable patients referred to community programmes 14, 15, 16. In extant studies, receiving clinic nurses were provided with additional training in the context of a clinical trial, the sickest patients were still cared for by physicians, and a substantial fraction of patients refused the referral 12, 14. Strategies for evaluating linkage to and retention in community clinic care under “real world” programmatic conditions will prove invaluable for designing programmes that maximize continuity of clinical HIV care following transfers.

Data from the National Health Laboratory Service (NHLS), South Africa's repository for all public sector laboratory measurements, have previously been used to evaluate the effectiveness of certain government‐funded HIV‐programmes 17, 18, 19, to identify patterns of the country's TB epidemic 20, 21, and to determine cancer incidence rates among HIV‐infected patients 22. While we have previously assessed the completeness and accuracy of the NHLS Corporate Data Warehouse (CDW) and have validated the NHLS CDW as a reliable data source 23, the use of NHLS data to evaluate the outcome of patient transfers has been limited 24. Despite that, it has been shown to be an effective tool for more accurately estimating lost to follow‐up for women transferring to postpartum care 25, 26. With its large size and comprehensive coverage (>80%) of the population 17, 27, the NHLS CDW could serve as an important tool for tracking patients following healthcare facility closures or other care transfers. As programmes across South Africa continue to transfer patients from non‐governmental organizations to government‐funded, community clinics, identifying methods to inform the success of these transfers is critical.

Following a large‐scale transfer from a physician‐run, hospital‐based, PEPFAR‐funded HIV programme in Durban to nurse‐led, community‐based clinics, we sought to demonstrate the utility of NHLS data to evaluate success of transfer and to determine predictors of slower re‐linkage to care.

## 2 Methods

### 2.1 Study site

McCord Hospital was a semi‐private, general hospital in KwaZulu‐Natal serving a predominantly urban population from the greater Durban area. The Sinikithemba HIV clinic at McCord, which became a PEPFAR‐funded site in 2004, was an integral part of the South African ART scale‐up and initiated over 10,000 patients on ART 28. Sinikithemba served a predominantly African, Zulu‐speaking population. Patients were seen by both nurses and doctors who were available for daily consultations and took on medically complex cases. The clinic was considered a local Center of Excellence and had a monitoring and evaluation team and an electronic medical record. Due to loss of PEPFAR funding, the clinic closed in June 2012.

All patients who returned to the clinic for clinical appointments, laboratories or pharmacy refills to Sinikithemba between 12 March 2012 and 30 June 2012, the “transfer period,” were referred for transfer to one of 171 different clinics in the Durban area. Patients taking first‐line ART were transferred to primary healthcare clinics (PHC); those taking second‐line line ART were transferred to community health clinics (CHC), which provide a higher level of services than PHCs. Patients with comorbidities requiring medications not on the South African Essential Drug List were referred to hospital‐based clinics. Data collected by the clinic at the time of transfer included sex, age, most recent CD4 count and viral load (VL) prior to transfer, and the type of clinic to which the participant were referred. We have previously reported on the Sinikithemba transfer process evaluating linkage to the initial transfer clinic visit and patient attitudes about their transfer experience using telephone surveys combined with validated clinic visits 28, 29. The current study seeks to assess linkage to HIV care post‐transfer using availability of CD4 or VL testing within approximately three years of transfer as a proxy for successful transfer.

### 2.2 Study population

The study population included adults on ART who visited the Sinikithemba HIV clinic during the transfer period. Patients <18 years old on 30 June 2012 were excluded, as we focused on the adult transfer process. For the post‐transfer linkage assessment, we excluded patients who were referred to a private doctor, because we did not expect these individuals to have laboratory data in the NHLS system. Participants provided verbal consent for study participation at the time of transfer. The study protocol was approved by the McCord Hospital Research Ethics Committee (Durban, South Africa) and the Partners Human Research Committee (2012‐P‐001,122/1, Boston, MA).

### 2.3 National Health Laboratory Service

The National Health Laboratory Service was established in 2001 and supports national and provincial health departments in South Africa. It is the largest diagnostic pathology service in the country, providing laboratory and related public health services to over 80% of the population through a national network of 265 laboratories 17. The NHLS performs all public sector CD4 count and VL monitoring and maintains a Corporate Data Warehouse (CDW) that serves as a national repository for laboratory data from the public sector. Healthcare workers at each public health facility complete laboratory requisition forms which accompany each sample submitted to the CDW. All data, including patient identifiers, name of facility, date of sample and tests requested, are sent to the CDW and are captured electronically by the NHLS information system in real time. We have previously evaluated the completeness and accuracy of the NHLS CDW using a novel data crossmatching method 23.

### 2.4 Data collection and processing

We sought to estimate the cumulative frequency of linkage using evidence of post‐transfer CD4 count and VL after 30 June 2012. We sent a list of all 4257 McCord Hospital transfer patients with corresponding identifiers (patient ID, first name, surname, sex, date of birth, South African ID) to the NHLS to obtain laboratory records within approximately three years of the transfer (the last NHLS records extracted were in June 2015). To assist with the matching process, we also sent the last known CD4 count and VL values and dates recorded in the electronic medical record at McCord Hospital. From the original 4257 patient list, duplicated patient IDs (n = 12), patients <18 years old on 30 June 2012 (n = 337), and patients referred to private clinics (n = 15) were removed from the data sets prior to matching, resulting in a cohort of 3893 transfer patients for potential matching with NHLS laboratory data. Although laboratory monitoring recommendations have changed over time, laboratory monitoring of some kind (CD4 or VL or both) have been recommended every six or twelve months in South Africa for the duration of the study period.

We assessed deaths by cross‐matching patients with the South African National Population Register using South African Identification Numbers in May 2014. Valid South African Identification Numbers were available for 94% of patients. More than 90% of deaths nationwide are captured in this register 30.

### 2.5 Assessment of cumulative frequency of linkage after transfer

As we reported previously, after extensive efforts assessing the quality of subject matching for data provided by the NHLS, we determined that 99.7% of the individuals were correctly matched 23. Based on this result, we previously recommended that matches from NHLS be accepted without extensive review, and have followed this approach in the current analysis 23. Patients who had at least one NHLS record, whether CD4 count or VL, within approximately three years after the end of the transfer period were considered re‐linked. We also assessed re‐linkage rates at six months, one year and two years after the end of the transfer period.

### 2.6 Statistical analysis

Standard life table methods were used in the analysis. Proportions linked at various time periods after the end of the transfer period were estimated using Kaplan‐Meier methods. We used a Cox proportional hazards model to examine the hazard ratio of slower time to re‐linkage (having no post‐transfer measurements in the CDW over time). Predictors considered included sex, age, CD4 count at transfer (three levels: <200/μL; 200 to 500/μL; and >500/μL), virological suppression pre‐transfer (three categories: suppressed, defined as NHLS coding of <400 copies/mL; not suppressed; and no VL data available), duration on ART prior to transfer (three categories: <3 months; three months to one year; and >1 year) and type of transfer facility. We excluded a total of 44 individuals with missing values for CD4 (3 individuals), ART duration (23 individuals), or missing location of future care (21 missing); several individuals were missing data on more than variable. We retained missing VL as a separate category, due to the large number of missing records (298). We calculated hazard ratios (HR) with 95% confidence intervals (CI) for each individual factor. We then developed a multivariable model including all factors other than VL that were statistically significant in the univariate analysis and calculated adjusted hazard ratios (aHR). The goal of the model was to identify factors associated with higher risk of failure to re‐link to care after clinic transfer to help guide programmes when considering which patients may be higher risk for LTFU post transfer. Statistical analyses were performed using SAS software, version 9.4 (SAS Institute, Cary, NC).

## 3 Results

### 3.1 Cohort characteristics

Of the 3893 participants included in the analysis, 41% of the cohort was male and median age was 39 (interquartile range (IQR) 34 to 46) (Table 1). The majority of patients had CD4 counts above 200/μL at transfer (<200/μL 16%, 200 to 500/μL 56%, >500/μL 29%) and 98% of patients with available VL data were virologically suppressed. As 298 patients (8%) did not have a VL available, however, the proportion known to be suppressed is only 90%. Among all participants, over 80% had been on ART for more than a year upon transfer (<3 months 5%, three months to one year 13%, >1 year 82%). Most patients were transferred to public sector clinics (PHC 67%, CHC 21%, hospital 12%). Fifteen patients (0.4%) were assigned to private doctors and were excluded as we did not expect that they would have laboratory tests through the NHLS. Eighty‐four patients (2.2%) are known to have died after transfer but are included in our population for assessing re‐linkage to care after transfer based on the availability of NHLS laboratory testing after the transfer period.

**Table 1:** Characteristics of patients transferred from McCord Hospital to community‐based clinics in Durban, South Africa

Patient characteristic	Total N = 3893
Sex (male), N (%)	1586 (41)
Age, median (IQR)	39 (34 to 46)
Age<30, N (%)	406 (10)
CD4 count at transfer, N (%)^a^	
<200/μL	606 (16)
200 to 500/μL	2164 (56)
>500/μL	1120 (29)
Viral load, % known suppressed at transfer, N (%)^b^	3514 (90)
Duration of ART treatment, N (%)^c^	
<3 months	186 (5)
3 months to 1 year	523 (13)
>1 year	3161 (82)
Location of future care, N (%)^d^	
Primary healthcare clinic	2609 (67)
Community health clinic	811 (21)
Hospital‐based clinic	452 (12)
Number of known deaths in the cohort, N (%)	84 (2)

ART, antiretroviral therapy.

^a^Based on the last CD4 count value taken at McCord excluding three missing values; ^b^based on the last viral load value taken at McCord, with 298 missing values treated as not suppressed; ^c^based on the total time in years between the ART initiation date and the transfer date excluding 23 missing values; ^d^Excluding 21 “unknown” locations of future care.

### 3.2 Cumulative frequency of linkage to care over time

**Figure 1:**
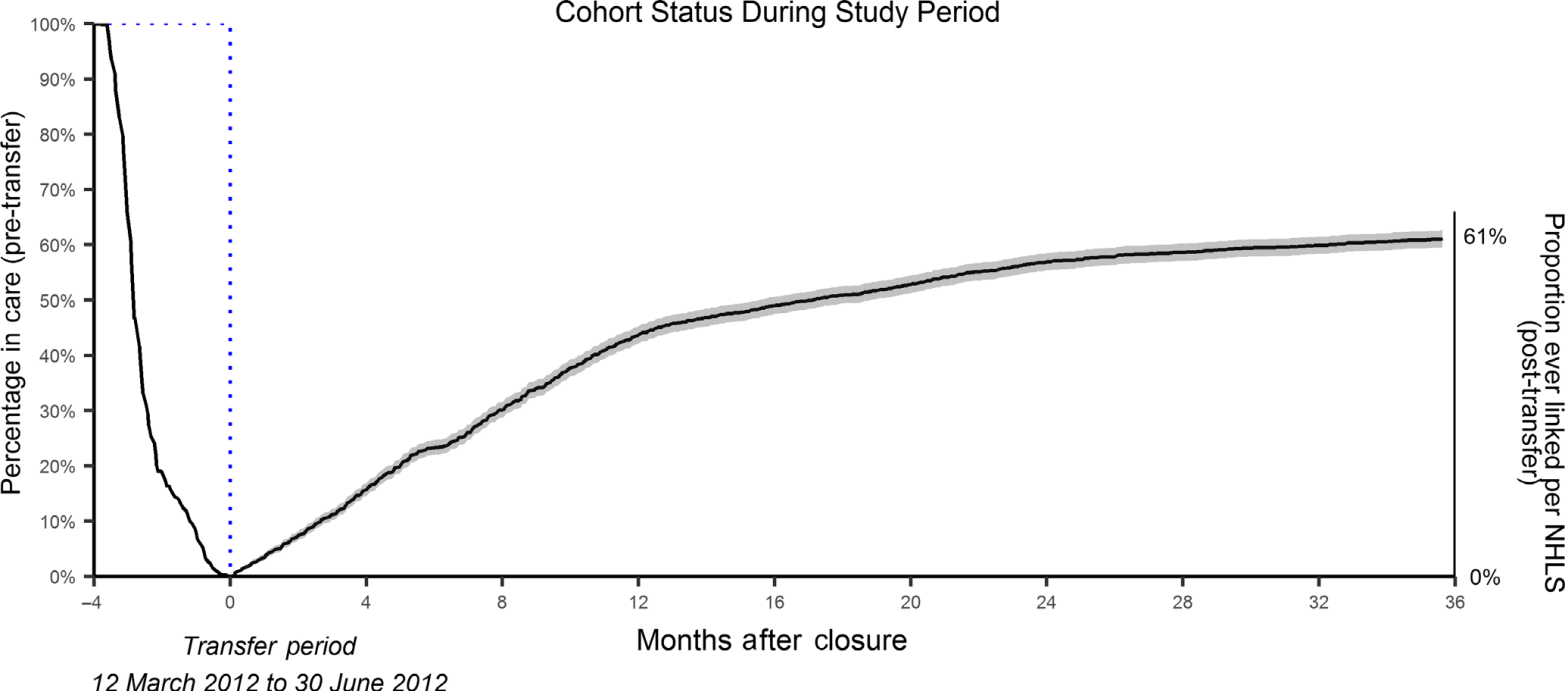
The proportion of individuals in care prior to transfer or linked to care after transfer The left axis shows the proportion of the total population in care from the start of the study, while the right axis shows the proportion of those who have re‐linked among the total population by the end of the study period. We assumed all 3893 individuals were in‐care on 1 March 2012, shortly before the transfers began. The number in care then decreased as they completed their final visit at the initial site. The proportion having a CD4 count or viral load measurement in the National Health Laboratory System (NHLS) Corporate Data Warehouse (CDW) over time after the end of the transfer period is the solid line. Time in months is shown along the horizontal axis, with time=0 the end of the transfer period. Confidence bands (shaded lines) depict the 95% Hall‐Weller confidence interval, which provides an approximate 95% coverage throughout the entire re‐linkage period. No confidence interval is shown for the data prior to transfer or for the early period (approximately first month) after transfer. Twenty‐three percent of the original population were linked within six months of the end of the transfer period, 44% were linked within one year, 57% were linked within two years, and 61% were linked within approximately three years.

Among the 3893 patients transferred, 61% (2377) of these patients were re‐linked to care by the end of our study period (Figure 1). Twenty‐three percent were linked within six months of the end of the transfer period, 44% were linked within one year of the transfer period and 57% were linked within two years of the end of the transfer period. Among the 84 patients known to have died, 33 (39%) had NHLS laboratory data after transfer and before death.

### 3.3 Predictors of slower re‐linkage to care

In the unadjusted analyses, male sex (hazard ratio 1.19 (1.09 to 1.30)), age <30 (compared to age ≥30 years, hazard ratio 1.27 (1.10 to 1.47)), CD4 count <200/μL (compared to >500/μL, hazard ratio 1.38 (1.20 to 1.58), and shorter duration of ART (<3 months hazard ratio 3.96 (2.90 to 5.40) and three months to one year hazard ratio 1.35 (1.19 to 1.54), both compared to >1 year) were associated with slower re‐linkage over time (Table 2). Missing VL data were a significant predictor of slower linkage (hazard ratio 3.31 (2.63 to 4.17)) but lack of suppression was not an important predictor (hazard ratio 1.12 (0.83 to 1.51)). The type of clinic to which participants were referred at transfer was also not associated with failure to re‐link.

**Table 2:** Predictors of not having post‐transfer measurement data in the NHLS CDW

Patient characteristic	Linked N = 2353 (61%)	Not linked N = 1496 (39%)	Hazard ratio (95% CI)	Adjusted hazard ratio model (95% CI)^a^
Sex (male), N (%)	901 (38)	672 (45)	1.19 (1.09 to 1.30)	1.20 (1.10 to 1.31)
Age <30, N (%)	210 (9)	187 (13)	1.27 (1.10 to 1.47)	1.12 (0.96 to 1.30)
CD4 count at transfer, N (%)				
<200/μL	310 (13)	294 (20)	1.38 (1.20 to 1.58)	1.04 (0.90 to 1.20)
200 to 500/μL	1329 (57)	807 (54)	1.07 (0.97 to 1.18)	0.98 (0.89 to 1.08)
>500/μL	714 (30)	395 (26)	ref	ref
Viral load, N (%)				
Suppressed at transfer	2233 (95)	1244 (83)	ref	–
Not suppressed at transfer	45 (2)	33 (2)	1.12 (0.83 to 1.51)	–
Missing	75 (3)	219 (15)	3.31 (2.63 to 4.17)	–
ART duration, N (%)				
<3 months	41 (2)	144 (10)	3.96 (2.90 to 5.40)	3.80 (2.77 to5.21)
3 months to 1 year	270 (11)	246 (16)	1.35 (1.19 to 1.54)	1.32 (1.15 to 1.51)
>1 year	2042 (87)	1106 (74)	ref	ref
Location of future care, N (%)				
Primary healthcare clinic	1567 (67)	1029 (69)	ref	–
Community health clinic	510 (22)	295 (20)	0.94 (0.85 to 1.04)	–
Hospital‐based clinic	276 (12)	172 (11)	0.92 (0.81 to 1.06)	–

A total of 44 individuals (24 linked; 20 not linked) were excluded for missing one or more of CD4, ART duration, or location of future care. ART, antiretroviral therapy; CDW, Corporate Data Warehouse; NHLS, National Health Laboratory Service; ref, reference group.

a Model adjusted for sex, age, CD4 count at transfer, and time on ART prior to transfer.

#### 3.3.1 Multivariable model

Because only lack of VL data, strongly associated with shorter duration on ART (data not shown) was a significant predictor of slower linkage, VL was not included in the multivariable model. Males were more likely to be unlinked (adjusted hazard ratio 1.20 (1.10 to 1.31)) but age and CD4 count were no longer significant predictors (both *p* > 0.10) (Table 2). Being on ART for a shorter duration at transfer (<3 months, adjusted hazard ratio 3.80 (2.77 to 5.21); three months to one year, adjusted hazard ratio 1.32 (1.15 to 1.51), each compared to >1 year) also increased the hazard of not being linked.

## 4 Discussion

We assessed CD4 count or VL testing as a proxy for successful transfer of HIV care following a large‐scale transfer from McCord Hospital in Durban to nurse‐led, community‐based clinics by obtaining patient data from the NHLS CDW. We found that 2377 of the 3893 patients eligible for post‐transfer assessment (61%) had evidence of a post‐transfer, follow‐up visit based on NHLS CD4 count and VL data at approximately three years. We consider 61% to be a low estimate, based on the 221 who reported transferring to a private doctor in our prior study 28. These findings are consistent with systematic reviews estimating 70% retention in care at 24 months among patients on ART in sub‐Saharan Africa 31, 32. A more recent review focusing on retention in care between 2008 and 2013 in South Africa estimated an 18‐month retention rate of 71% 33. While these rates are comparable with our estimate, these studies do not exclusively focus on cohorts of transferred or “down‐referred” patients who may face greater risks of disruption of care and loss to follow‐up. However, our estimate is also consistent with a study evaluating national retention in South Africa's HIV programme accounting for patient transfers estimating a 63% retention rate after six years 24 and with a study using NHLS data to assess postpartum transfers to general ART clinics, which found 65% of women linked and in care at 24 months 26.

In the multivariable analysis, male sex was a significant predictor of not having a post‐transfer record found in the NHLS CDW. Other factors that we evaluated (age, CD4 count at transfer and type of transfer facility) were not significantly associated with not having a post‐transfer record. These findings are consistent with our results from a previous study evaluating linkage to an initial transfer clinic visit, in which we found that age and most recent CD4 count were not associated with failure to be found at an assigned validation clinic 28. We previously estimated that 26% of patients continued care at clinics different than the assigned transfer clinic 28. Previous studies have shown that males typically have worse outcomes than females at every step of the HIV care cascade 34, 35. Male sex has emerged as a predictor of poor retention in care in a number of settings in sub‐Saharan Africa 32; our finding that male sex is associated with higher risk of not having a post‐transfer record in the NHLS CDW is consistent with these data.

In the ART duration adjusted model, spending <3 months on ART prior to transfer substantially increased the risk of not having a post‐transfer record. As detectable viral load was not a predictor of slower linkage, and missing VL information was strongly associated with transfer within three months of starting ART, ART duration, rather than viral suppression, may be a more appropriate measure for defining “high risk” transfer patients.

There are several limitations to using national laboratory data as a marker of cumulative frequency of linkage to care. Our results are an estimate of patients who re‐linked to care following transfer from McCord Hospital. This estimate is lower and may be more accurate than our previous estimate of 82%, which was based on patient self‐report of re‐linkage and validation of an initial clinic visit for a subset of patients 28. We have previously assessed the completeness and accuracy of the NHLS CDW by matching patient identifiers and pretransfer CD4 and VL test results from the McCord Hospital dataset to data returned by NHLS; we reported that NHLS did not find a match for 10% of the patients in the transfer cohort 23, which may have also affected the ability to match post‐transfer laboratories for a small proportion of patients. It is also possible that patients in care post‐transfer were classified as “not re‐linked” because they were not receiving guideline concordant CD4 count and VL monitoring at their new clinical site. Additionally, many more patients reported attending private sector clinics than were assigned to them at the time of transfer. If these private clinics used private laboratories, then these patients would not be expected to have CD4 count and VL data in the NHLS system even if they had linked to care. Removing these individuals from our potential pool would lead to a higher linkage rate. By focusing on laboratory data, we were unable to examine the reasons for gaps in linkage or to assess the quality of care received. The truncated ascertainment of outcomes and the fact that participants were on ART at transfer may explain the relatively low rate of mortality in the setting of a low rate of re‐linkage.

Despite the drawbacks of this methodology, this study has several important strengths. Using NHLS data can transcend the limitations of collecting and analysing data within individual programmes, which presents challenges such as differences in record‐keeping methods and marked variability in how patients are identified. The ability to accurately classify “lost to follow‐up” patients as having actually transferred care elsewhere is important for evaluating the quality of the national governmental ART programme, especially as known Centers of Excellence have been disbanded 36, 37. The NHLS CDW is centralized, comprehensive and offers wide coverage of public clinics across the country; it therefore represents a useful tool for helping track patients who change their service provider.

## 5 Conclusions

The cumulative frequency of linkage to care after large‐scale patient transfers is difficult to assess but has become increasingly important as PEPFAR funding in South Africa decreases and patient care transitions to public sector community clinics. We found that a national data set collected for clinical purposes may be used for evaluating linkage of individual patients within public, government‐funded clinics. This ability to track patients may not only aid in the evaluation of re‐linkage and retention outcomes following HIV‐clinic closures and patient transfers but may also serve as an effective method to study patient flow and movement more generally within the HIV care system in South Africa, such as following transfer to postpartum HIV care. Additionally, this study utilizes NHLS data in a novel way. While NHLS CDW data have been used in several cross‐sectional studies 17, 18, 19, 20, 21, they have only been used in one longitudinal cohort study involving South Africa's National Cancer Registry 22. This methodology may be useful in the assessment of similar large‐scale transfers in other sub‐Saharan African countries with PEPFAR‐funded clinics and national electronic laboratory databases 38. For those who fail to link after transfer, these data could also direct efforts to reinforce linkage and avoid the morbidity and mortality associated with interruptions in care.

## Competing interests

The authors have no competing interests to declare.

## Authors’ contributions

All authors have contributed significantly to this work and have reviewed and approved of this manuscript. IVB, Principal Investigator of this project, led the design and execution of this study as well as all stages of manuscript writing and preparation. MH and RAP led all data analysis efforts. MH initially helped to conduct the preliminary data analysis, while RAP collaboratively refined the analysis presented in the manuscript. RAP also contributed substantially and oversaw all method and analysis development. CC and JG both played significant roles in initial data collection and the procurement of records from McCord Hospital. SC also played a significant role in the procurement of CD4 and viral load records from the National Health Laboratory Services, which were used in the data crossmatch. EL, KAF, and RPW provided consultation during analysis development and contributed to manuscript editing and review. Additionally, EL provided biostatistical consultation. SCF, the Research Assistant, contributed significantly to manuscript writing, editing, and review.
